# What role does GPR65 play in the progression of osteosarcoma? Its mechanism and clinical significance

**DOI:** 10.1186/s12935-024-03216-5

**Published:** 2024-01-13

**Authors:** Jin Qi, Sihang Liu, Zhirui Zhang

**Affiliations:** 1 Department of Orthopedics, The First Affiliated Hospital of Wannan Medical College (Yijishan Hospital of Wannan Medical College), Wuhu, 241001 Anhui Province China; 2 Department of Pharmacy, The First Affiliated Hospital of Wannan Medical College (Yijishan Hospital of Wannan Medical College), Wuhu, 241001 Anhui Province China

**Keywords:** Osteosarcoma, GPR65, Immune escape, Biomarker Proliferation

## Abstract

**Background:**

GPR65 is a pH-sensing G-protein-coupled receptor that acts as a key innate immune checkpoint in the human tumor microenvironment, inhibiting the release of inflammatory factors and inducing significant upregulation of tissue repair genes. However, the expression pattern and function of GPR65 in osteosarcoma (OS) remain unclear. The purpose of this study was to investigate and elucidate the role of GPR65 in the microenvironment, proliferation and migration of OS.

**Methods:**

Retrospective RNA-seq data analysis was conducted in a cohort of 97 patients with OS data in the TAEGET database. In addition, single-cell sequencing data from six surgical specimens of human OS patients was used to analyze the molecular evolution process during OS genesis. Tissues chips and bioinformatics results were used to verify GPR65 expression level in OS. MTT, colony formation, EdU assay, wound healing, transwell assay and F-actin assay were utilized to analyze cell proliferation and invasion of OS cancer cells. RNA-seq was used to explore the potential mechanism of GPR65’s role in OS.

**Results:**

GPR65 expression was significantly low in OS, and subgroup analysis found that younger OS patients, OS patients in metastatic status, and overall survival and progression free survival OS patients had lower GPR65 expression. From ScRNA-seq data of GSE162454, we found the expression of GPR65 is significantly positively correlated with CD4 + T cells CD8 + T cells and OS related macrophage infiltration. Verification experiment found that silencing the expression of GPR65 in osteosarcoma cells U2OS and HOS could promote the proliferation and invasion process, RNA-seq results showed that the role of GPR65 in OS cells was related to immune system, metabolism and signal transduction.

**Conclusion:**

The low expression of GPR65 in OS leads to high metastasis rate and poor prognosis in OS patients. The suppression of immune escape and inhibition of proliferation may be a key pathway for GPR65 to participate in the progression of OS. The current study strengthens the role of GPR65 in OS development and provides a potential biomarker for the prognosis of OS patients.

**Supplementary Information:**

The online version contains supplementary material available at 10.1186/s12935-024-03216-5.

## Introduction

Osteosarcoma (OS) is the most common primary malignant bone tumor in children and adolescents, and recurrence and metastasis (mostly lung metastasis) are the main reasons for unsatisfactory treatment of OS [1, 2]. The activity imbalance of osteoblasts and osteoclast in the microenvironment of OS and the different types of malignant differentiation of tumor stem cells lead to the pathological classification of OS into osteoblastic OS, chondroblastic OS, fibroblastic OS, mixed OS, etc [3, 4]. PH is a key regulator of osteoblast and osteoclast activity [5]. GPR65 (TDAG8) is a glycosphingolipid psychoactive amine (d-nenenebb galactose group-β-1,1’-sphingosine) receptor which has been proved to be a pH sensitive G protein coupled receptor [6, 7]. Ovariectomized mice in the absence of GPR65 showed increased bone resorption, increased number of osteoclast, and increased activity of osteoclast, leading to excessive bone resorption [8]. The activation of GPR65 inhibits calcium absorption in osteoclast, thereby improving bone density [9]. It can be seen that the expression of GPR65 plays a vital role between osteogenesis and osteoclast.

Importantly, recent studies have found that GPR65 could be used as a key cancer immune checkpoint inhibitor in human tumor microenvironment, which could inhibit the release of inflammatory factors and induced significant up-regulation of tissue repair genes [10, 11]. GPR65 is a member of the proton-sensing G protein-coupled receptor family, which is closely related to tumor microenvironment (TME) [12]. TME is composed of Extracellular matrix (ECM), stromal cells and immune cells (including T lymphocytes, B lymphocytes, tumor-associated macrophages, etc.) [13]. The composition of TME has been found to influence immune checkpoint blockade responses [14]. In the OS microenvironment, does GPR65 play an immunosuppressive role and promote the immune escape of OS? Or is it because GPR65 is strongly expressed in lymphoid tissue (tumor suppressor factor), its activation may represent a potential anti-tumor biomarker? It is currently unclear. Therefore, understanding the regulation and molecular function of GPR65 may indicate the new potential therapeutic target and prognosis predictor for OS.

This study first analyzed the GPR65 expression of 97 patients with OS from TARGET database, and deeply analyzed the potential molecular network of GPR65 in OS cells and its role in biological processes. The experiment further verifies the role of GPR65 in OS. Our study found that GPR65, different from other types of cancer (colon cancer, pancreatic cancer, etc.), has a new expression feature in OS patients, and reveals that the low expression of GPR65 indicates poor prognosis in OS patients.

## Materials and methods

### Data collection

In this study, transcriptome data and clinical data (101 TARGET-OS gene expression data) of OS were downloaded from TARGET database. After deleting missing data and data without follow-up records, data from 97 patients with OS were retained for final research analysis. Detailed information can be found in the supplementary file 1. The expression of GPR65 in various tumor tissues and normal tissues was analyzed using Gene Expression Profiling Interaction Analysis (GEPIA; http://gepiagepia.Cancer-pku.cn/) Analysis. Due to the fact that the GEPIA database only contains GPR65 expression data information for sarcomas, application of Gene Chip Technology to detect the expression of GPR65 in both OS and normal tissues.

### Difference analysis

The gene expression data of different subgroups of transcriptome of OS patients were mapped with the R software ggplot2 package, and two samples were t-tested. Using the R software pROC package to draw ROC curves.

### Functional enrichment analysis

To conduct GO enrichment analysis and Kyoto Encyclopedia of Genes and Genomes (KEGG) pathway analysis, GPR65 related genes, or a characteristic gene list of the cell cluster were obtained from the Database for Annotation, Visualization, and Integrated Discovery (https://david.ncifcrf.gov/) download. The official genetic symbol was selected as the identifier, and Homo sapiens was selected as the spec in ies. In this study, the top six results (in ascending order of P value, *P* < 0.05) for biological process (BP), cell component (CC), and molecular function (MF) analysis were performed using R software DOSE, clusterProfiler, and enrichment software packages.

### Relationship between GPR65 expression and survival prognosis

COX proportional hazards model was employed to examine the association among GPR65 mRNA expression and survival prognosis. IBM SPSS software (21.1 version) were used. Kaplan Meier analysis of GPR65 mRNA expression and OS survival prognosis was conducted using online analysis platform on 98 OS patients in TARGET database (https://www.aclbi.com/static/index.html#/target).

### Single-cell RNA sequencing (ScRNA-seq)

ScRNA-seq data of GSE162454 was downloaded from the Gene Expression Omnibus (GEO) database(http://www.ncbi.nlm.nih.gov/geo/), including 6 samples of human primary OS. The scRNA-seq date was firstly converted to Seurat objects. The quality control of scRNA-seq data, “NormalizeData”, principal component analysis (PCA), the “Find All Markers” function and annotate the cell subpopulations of the different clusters were performed by the R Seurat package which have been described in detail in previous study [15].

### Cell lines and cell culture

OS cell lines U2OS and HOS were obtained from Procell Life Science&Technology Co.,Ltd (Wuhan, China). U2OS was maintained in McCoy’s 5 A (Procell, China) and HOS was maintained in DMEM (Procell, China) supplemented with 10% fetal bovine serum (FBS) (Yeasen, Shanghai, China), 100 U/mL penicillin, and 100 µg/mL streptomycin. Cells were cultured at 37 °C with 5% CO_2_.

### Cell transfection

The cells were transfected with the siRNA/plasmids using Lipofectamine™ 3000 Transfection Reagent (Thermo Fisher Waltham, MA, USA) according to the manufacturer’s instructions. The following siRNAs were used in this study: GPR65 siRNA#1: CAGUGGUCUACAUAUUUGUTT; GPR65 siRNA#2: GAAUCCGUCUUUAACUCCATT; and the control siRNA was UUCUCCGAACGUGUCACGUTT.

### Cell viability assay

The U2OS and HOS cells were cultured at a density of 6000 cells/well in 96-well plates and then transfected with Flag-GPR65 plasmids or GPR65 siRNA. Continue cultivation for 36 h and then 20 µl of 5 mg/ml MTT was added. After 2 h, the medium was discarded and 150 µl DMSO was added. The absorbance values of each sample were measured at 490 nm using a spectrophotometer (Elx800, BioTEk instrument, USA).

### Colony formation assay

The U2OS and HOS cells (1500–2000/well) were passaged in 12-well plates and treated with transfection. After 7 to 10 days of culture, cells were fixed with 4% paraformaldehyde for 15 min and stained with crystal violet, cell colonies were photographed using a camera.

### EdU assay

The U2OS and HOS cells (5 × 10^4^ cells/well) were plated in 24-well plates. The EdU incorporation assay was the BeyoClick™ EdU Cell Proliferation Kit with Alexa Fluor 594 (Beyotime Biotechnology, Shanghai, China), the experiment was performed as described in the previous published article [16].

### F-actin filaments assay

U2OS or HOS cells (5 × 10^4^ cells/well) were passaged in 24-well plates and transfected with Flag-GPR65 or siRNA. After incubating for 36 h, the cells were fixed with 4% paraformaldehyde and then 0.3% Triton X-100 was permeabilized. Add enough phalloidin (green fluorescence) staining solution into cells for 30 min. Images were captured with a fluorescence microscope.

### Wound healing assay

U2OS or HOS cells (1 × 10^5^ cells/well) were seeded in 6-well plates, the next day scratched using pipette tips. Subsequently, cells were transfected with Flag-GPR65 or siRNA. Migrated cells at o and 36 h were monitored and captured using microscopy.

### Transwell migration assay

After the transfection, U2OS or HOS cells were plated at a density of 2.5 × 10^4^ cells/ml in 400 µl 1% medium in the upper chamber, while the lower chamber contained 600 µl normal medium. After 36 h co-culture, the upper membrane cells were fixed in 4% paraformaldehyde and then stained with 1% crystal violet. Images were captured by inverted optical microscopy.

### RNA extraction and quantitative reverse transcription PCR (qRT-PCR)

Total RNA was extracted using according to the manufacturer’s instructions (YiFeiXue, Nanjing, China). qRT–PCR was performed using the 2×SYBR Green qPCR Mix (Shandong Sparkjade Biotechnology Co., Ltd., Shandong, China). The primer sequences are listed in the Table 1.

**Table 1:** List of oligonucleotides used for qPCR analyses

Target name	Sequence (5’- 3’)
GPR65 (Human)	TCACCATCCTGATCTGCAAC
	TTTTCCTTGTTTTCCGTGGC
E-cadherin (Human)	CGAGAGCTACACGTTCACGG
	GGGTGTCGAGGGAAAAATAGG
N-cadherin (Human)	AGCCAACCTTAACTGAGGAGT
	GGCAAGTTGATTGGAGGGATG
Vimentin (Human)	GACGCCATCAACACCGAGTT
	CTTTGTCGTTGGTTAGCTGGT
Snail (Human)	TCGGAAGCCTAACTACAGCGA
	AGATGAGCATTGGCAGCGAG
Twist (Human)	GTCCGCAGTCTTACGAGGAG
	GCTTGAGGGTCTGAATCTTGCT
Zeb1 (Human)	TTACACCTTTGCATACAGAACCC
	TTTACGATTACACCCAGACTGC
Zeb2 (Human)	CAAGAGGCGCAAACAAGCC
	GGTTGGCAATACCGTCATCC

### RNA-sequencing and analysis

The U2OS cells were transfected with Flag-GPR65 and control plasmid respectively, total RNA was extracted using according to the manufacturer’s instructions (YiFeiXue, Nanjing, China). Shanghai Major-bio Biopharm Biotechnology (Shanghai, China) performed the transcriptome sequencing and analyses. And the data were analyzed on the Majorbio Cloud Platform (www.majorbio.com). Differentially expressed genes (DEGs) with *P* < 0.05 were identified [16].

### Western blot analysis

Total proteins were extracted from synovial tissues and cells by RIPA lysis buffer (Beyotime Institute of Biotechnology), the extracted protein levels were determined by BCA assay (Yeasen Biotech, Shanghai, China). Equal amounts of total protein were separated by SDS-PAGE, followed by transfer to the PVDF membranes. After blocked by 5% skim milk, the membranes were incubated with primary antibodies at 4 ◦C overnight. And incubated with the peroxidase-conjugated secondary antibody for 1 h the next day. All membranes were imaged with ECL super (Sparkjade, Shandong, China). The following antibodies were used: GPR65 (Cat#ER1910-13, Huabio, Hangzhou, China); GAPDH (Cat#AP0063, Bioworld, Nanjing, China); E-cadherin (Cat#R22490, Zen Bioscience, Chengdu, China); N-Cadherin (Cat# R23341, Zen Bioscience, Chengdu, China); Vimentin (Cat#R22775, Zen Bioscience, Chengdu, China).

### Statistical analysis

Expression differences were calculated using R software (version4.2.2), IBM-SPSS software (version21.1). The correlation between GPR65 and immune processes was determined by Pearson correlation analysis for Gene set variation analysis (GSVA). The prognostic value was evaluated by Kaplan-Meier and COX analysis. Gene ontology (GO) was performed in the DAVID portal website (https://david.ncifcrf.gov/summary.jsp). The correlation was tested by Pearson correlation analysis. Data are shown as the mean ± standard deviation of at least three independent experiments. Statistical significance was set at *P* ≤ 0.05.

## Result

### The expression and distribution characteristics of GPR65 in OS patients

GEPIA analysis found that GPR65 expression was higher in GBM, LAML, and KIRC patients, while GPR65 expression was lower in LUAD, LUSC, and THYM tissues. Compared with normal tissue, higher expression of GPR65 was observed in sarcoma tissue (supplementary Fig. 1). Although GEPIA describes sarcoma tissue more generally, it is necessary to analyze the expression of GPR65 in a single OS tissue. In TARGET-OS database, the distribution characteristics of OS patients with GPR65 divided into high expression group and low expression group according to the average value (the average expression of GPR65 is 6.884). There were 42 OS patients with GPR65 expression values less than 6.884, including 25 males and 17 females. There were 55 patients with GPR65 expression values greater than 6.884, including 32 males and 23 females. There are a total of 74 patients under to 18 years old, and 23 patients over 18 years old. Due to the missing survival status information of two OS patients, this study included ninety-five OS patients, including 58 surviving patients and 37 dead patients. There were 27 metastatic patients and 70 non-metastatic patients.

**Fig. 1:**
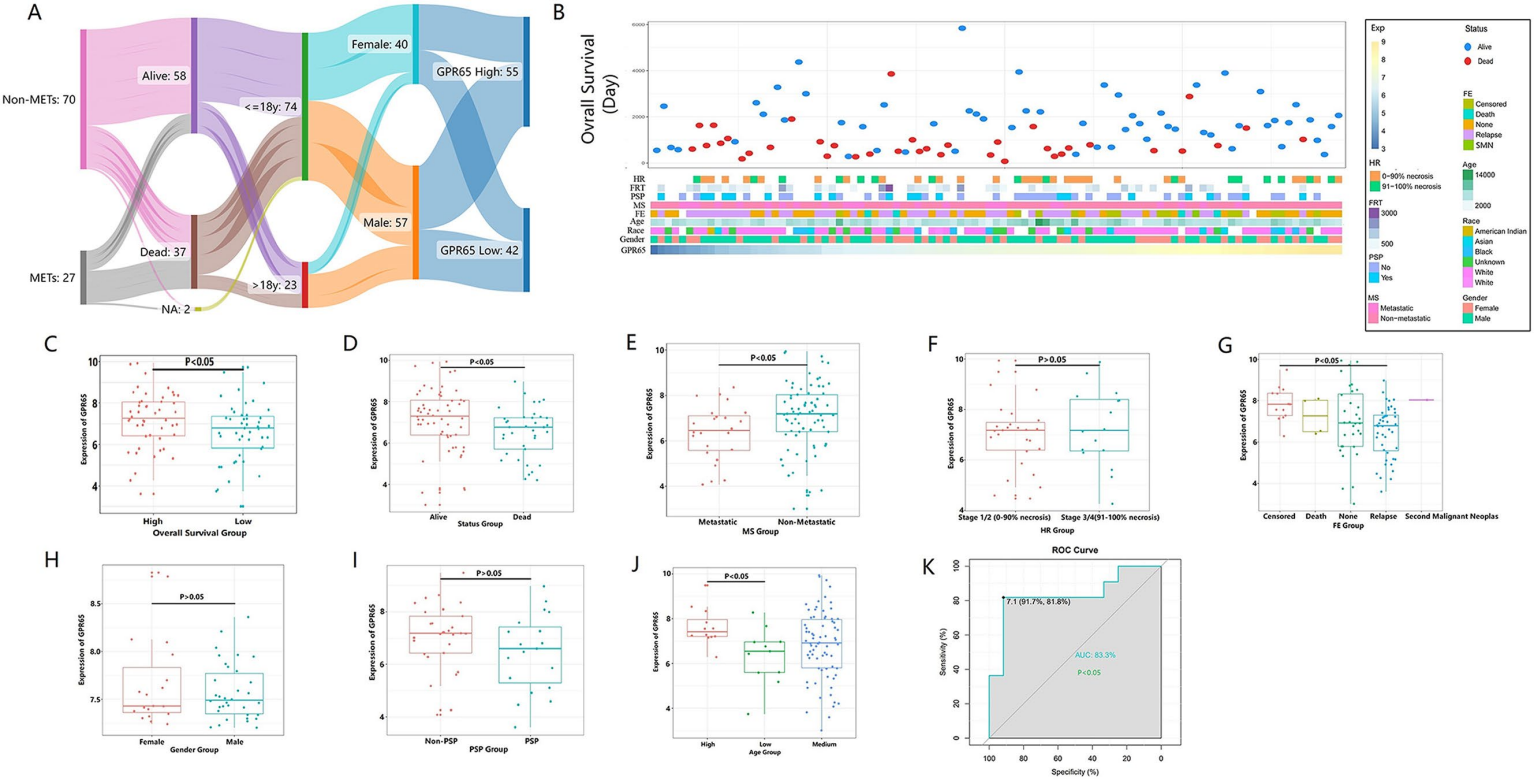
Association between GPR65 and clinicopathological characteristics of OS. (**A**) The expression of GPR65 shows asymmetric distribution in different groups of OS patients in Sankey diagram. (**B**) The landscape of GPR65-related clinicopathological significances of OS in TARGET database. (**C-J**) T-test detection of differential mRNA expression of GPR65 in different subgroups (such as overall survival rate, survival status, MS, HR, FE, gender, PSP, age). (**K**) In the TARGET database, the receiver-operating characteristic (ROC) curve shows high expression specificity of GPR65 in the subtypes of elderly OS patients

From the Sankey diagram, it could be seen that patients in the high and low expression groups of GPR65 exhibited asymmetric distribution in terms of gender, age, survival status, and metastasis status (Fig. 1A). Different levels of GPR65 expression indicated different clinical and pathological characteristics in OS patients. In the TARGET database, as the expression level of GPR65 increased, there was an asymmetric distribution in patient age, gender, race, HR, FRT, PSP, MS, FE and survival status (Fig. 1B). Further analysis revealed that as the survival time of patients increased, the expression of GPR65 showed an increasing trend. There was a statistically significant difference in GPR65 expression between OS patients with survival time less than 3 years and those with survival time greater than 3 years (Fig. 1C). The expression of GPR65 in OS patients in the dead group is lower than that in OS patients in the survival group (*P* < 0.05) (Fig. 1D). Consistent with the trend of GPR65 expression in patients’ status, the GPR65 expression in non-metastatic OS patients was higher than that in metastatic OS patients, and the difference between the two groups was statistically significant (*P* < 0.05) (Fig. 1E). There are many missing data in the pathological grade of OS patients (54 cases in total), with only 43 patients recorded. This may be the reason why there is no statistical difference in GPR65 expression between the pathological grade Stage1/2 (0–90% Necrosis) and Stage3/4 (91–100% Necrosis) groups (Fig. 1F). In the FE group, there was a decreasing trend in GPR65 expression among patients with FE recurrence (Fig. 1G), while there was no statistically significant difference in terms of patient’s gender, PSP, etc. (Fig. 1H, I). As is well known, osteosarcoma is more common in children, adolescents and young adults [14]. Coincidentally, in the TARGET database, GPR65 expression was higher in elderly OS patients (age > 20y) than in younger OS patients (age ≤ 10y) (*P* < 0.05) (Fig. 1J). Further using ROC curve to analyze the diagnostic value of GPR65 expression in terms of age in OS patients, the results showed that the area under the curve (AUC) of GPR65 gene in elderly osteosarcoma patients (age > 20 years old) was 83.30%, with statistical significance (*P* < 0.05) (Fig. 1K).

Overall, these results indicated that GPR65 expression was lower in younger OS patients (age ≤ 10y), metastatic osteosarcoma patients, and osteosarcoma patients with shorter survival time. GPR65 was expressed higher in elderly patients with OS (> 20 years of age), patients with non-metastatic OS, and patients with OS who had a longer survival time. These studies suggested that low expression of GPR65 was associated with poor prognosis in patients with OS.

### OS-associated GPR65 is associated with inflammatory response and osteoclast differentiation in OS

**Fig. 2:**
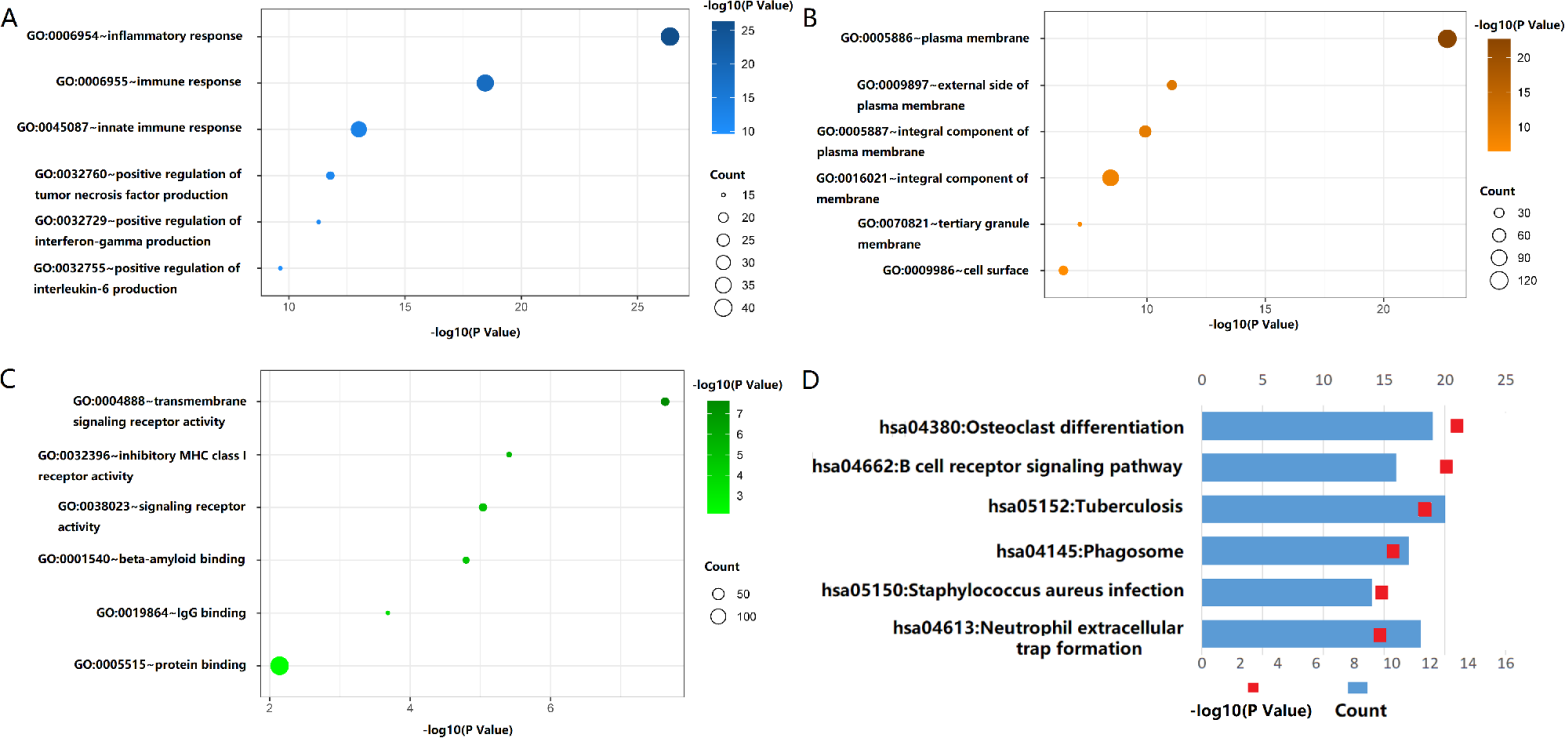
GPR65 is closely associated with inflammatory response and osteoclast differentiation of OS. (**A**) Biological processes analysis show GPR65 mainly participate in inflammatory response, innate immune response, etc. (**B**) Cellular components analysis show GPR65 mainly local in plasma membranes, integral components of membrane and cell surfaces. (**C**) Molecular functions analysis show GPR65 are related to transmembrane signaling receptor activity, inhibitory MHC-I receiver activity and signaling receptor activity. (**D**) KEGG analysis show that GPR65 is closely associated with osteoclast differentiation, B cell receptor signaling pathway, tuberculosis, neutrophil extracellular trap formation, etc

The biological process of GPR65 (GO-BP) is mainly enriched in the inflammatory response, immune response, and innate immune response pathways (Fig. 2A). The cellular components (GO-CC) are mainly enriched in plasma membrane, integral component of membrane, cell surface, etc. (Fig. 2B). The GPR65 molecular function (GO-MF) is mainly enriched in protein binding, transmembrane signaling receptor activity, inhibitor MHC class I receptor activity, signaling receptor activity/beta amyloid binding, and other functions (Fig. 2C). The results of KEGG pathway enrichment analysis showed that GPR65 was mainly enriched in the Osteoclast differentiation, B cell receptor signaling pathway, and Tuberculosis signaling pathways (Fig. 2D). These results suggested that the main biological function of OS associated GPR65 was related to the inflammatory immune response acting on the plasma membrane and the differentiation of osteoclast, which might be related to the involvement of GPR65 in OS immunity and bone repair after OS bone destruction under the OS immune microenvironment.

### OS associated GPR65 is positively correlated with tumor immune response, but negatively correlated with immunological memory process

**Fig. 3:**
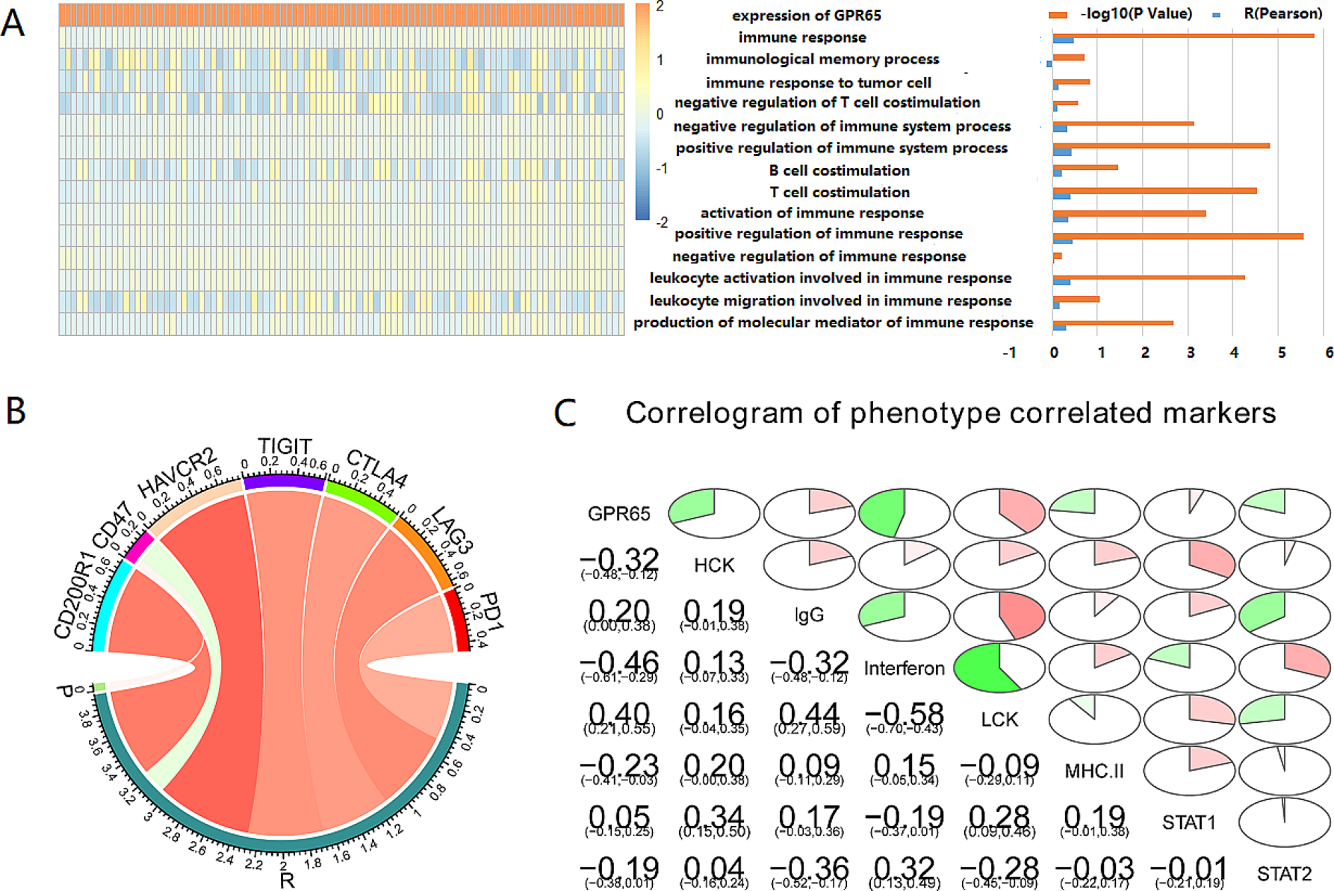
Correlation analysis between OS associated GPR65 and tumor immune response. (**A**) The heatmap showed the expression of OS associated GPR65 and the enrichment scores of immune functions of each patient in TARGET databases. The samples were arranged in ascending order of the expression of OS associated GPR65 (the red column represents P-value, and the blue column represents R-value). The horizontal axis (the depth or lightness of color) represents GPR65 enrichment scores, and the vertical axis has been marked in the middle position of Fig. 3A. (**B**) Pearson correlation between OS associated GPR65 and inhibitory immune checkpoints (The width of the band represented the R-value). (**C**) Pearson correlation analysis show correlation matrix of GPR65 and inflammatory-related meta genes. (The correlation coefficient was displayed in the bottom left corner. The correlation coefficient was expressed as the proportion of the pie chart. The red parts represent a positive correlation, while the green parts represent a negative correlation)

In order to enable the anti-tumor immune response to effectively kill tumor cells, the immune system initiates a series of immune responses (such as the release of chemokines and cytokines) and iteratively expands to eliminate the tumor [17]. However, in malignant tumor patients, lymphocytes, such as B cell, T cell and NK cell, may recognize antigens as self-antigens rather than foreign antigens, thus producing T regulatory cell responses rather than effector immune responses, or factors in the tumor microenvironment inhibit the function of effector lymphocytes, so that in tumor patients, tumor immune responses cannot play the best role [10]. Therefore, we examined the role of OS associated GPR65 activation in the immune response and corresponding cytokine characteristics of OS. The enrichment scores of different immune processes associated with OS GPR65 gene were analyzed using GSVA, and the results showed that OS associated GPR65 was highly correlated with immune response, especially with positive regulation of immune response and T cell co-stimulation (Fig. 3A). Collectively, the above findings revealed that GPR65 expression is involved in the tumor immune response of OS, but is negatively correlated with the immune memory of OS.

From the above analysis, it was found that GPR65 is correlated with tumor immunity. Therefore, further Pearson analysis was conducted to investigate the correlation between GPR65 and common cancer immune checkpoint inhibitors, such as CD200R1, CD47, HAVCR2, TIGIT, CTLA4, LAG3, and PD1. Research has found a correlation between GPR65 expression and the above immune detection points, especially HAVCR2/CD200R1 (*P* < 0.001, Fig. 3B). In addition, we selected seven immune system related meta gene cluster as markers of immune status. Calculate the GSVA enrichment score of 97 patients in the TARGET database, and then calculate the correlation between the seven immune system related inflammatory factor genes and OS associated GPR65 expression. The research results showed that OS associated GPR65 was negatively correlated with immune inflammatory factor genes such as HCK, Interferon, STAT2, etc., but positively correlated with IgG, MHC-II, STAT1, etc. (Fig. 3C).

**Fig. 4:**
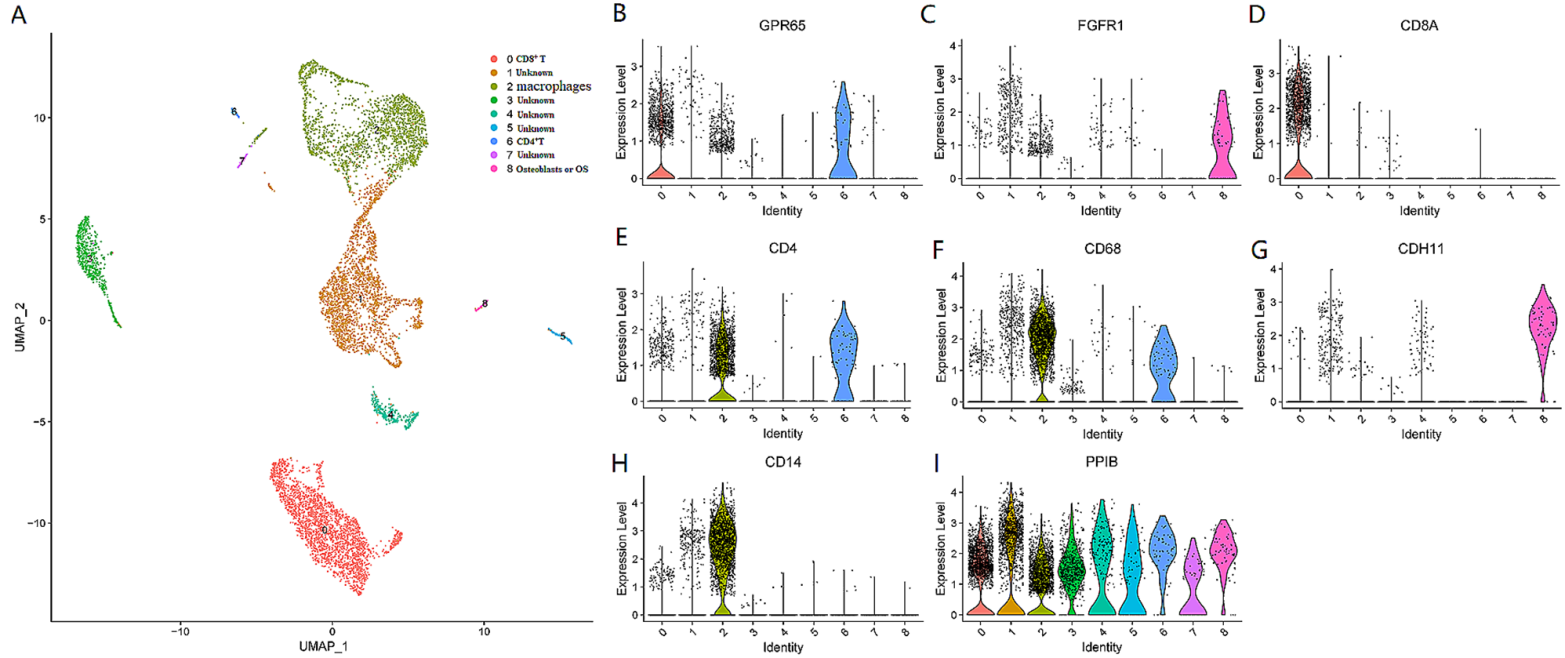
The expression pattern of GPR65 in OS microenvironment. (**A**) Distribution map of different cell Subclusters in OS Microenvironment from single-cell sequencing data. (**B**) GPR65 was mainly expressed on CD4 + T cells (cluster 6), CD8 + T cells (cluster 0), OS associated macrophage cells (cluster 2). (**C**&**G**) FGFR1 and CDH11 is mainly expressed on Osteoclast or OS cells (cluster 8). (**D**) CD 8A was mainly expressed on OS associated CD8 + T cells (cluster 0). (**E**) CD4 was mainly expressed on OS associated CD4 + T cells (cluster 6). (**F**) CD68 was mainly expressed on OS associated macrophage cells (cluster 2). (**H**) CD14 was mainly expressed on OS associated macrophage cells or monocytes (cluster 2). (**I**) PPIB was expressed in all cell clusters 0–8, especially in the cell cluster 0–3. The expression of above gene in different cells from GEO database (GSE162454)

### GPR65 is mainly expressed on OS associated macrophages and CD4 + T cells

Further analysis of ScRNA-seq from 6 cases of human OS identified different cell subpopulations, which were further clustered into 9 cellular metaclusters (Fig. 4A). Because CD4 is a marker for CD4 + T cells, CD8 is a marker for CD8 + T cells, and CD68 is a reliable marker for macrophages. CD14 is mainly expressed on the cell membranes of monocytes and macrophages. FGFR1 and CDH11 are markers of osteoclasts or osteosarcoma cells. From single-cell sequencing analysis, it was found that CD68 was expressed in clusters 2 and 6 (Fig. 4F), indicating that clusters 2 and 6 might be macrophages. In addition, CD14 was mainly expressed in cluster 2 cells (Fig. 4H), therefore, indicating that cluster 2 cells were macrophages. It was not difficult to see (Fig. 4E) that the 6 clusters of cells were CD4 + T cells. Cluster 0 represents CD8 + T cells (Fig. 4D). FGFR1 (Fig. 4C) and CDH11 (Fig. 4G) were mainly expressed on 8 clusters of cells, thus it could be determined that the 8 clusters were osteoblasts or osteosarcoma cells. PPIB was expressed in all cell clusters and was not a specific cell marker (Fig. 4I). However, the 1/3/4/5/7 cluster cells lack clear surface markers to determine the corresponding cell type. From Fig. 4B, it could be observed that GPR65 was mainly expressed in clusters 0, 2, and 6. In other words, GPR65 was mainly expressed on CD8 + T cells, CD4 + T cells, and tumor associated macrophages, but not on 8-cluster cells (osteoblasts or OS cells). Taken together, these data indicate that GPR65 may affect the function of OS associated macrophages, CD4 + T cells and CD8 + T cells in the OS microenvironment, and further affect OS cells proliferation.

### GPR65 is an independent prognostic factor for improved overall survival of OS patients

**Fig. 5:**
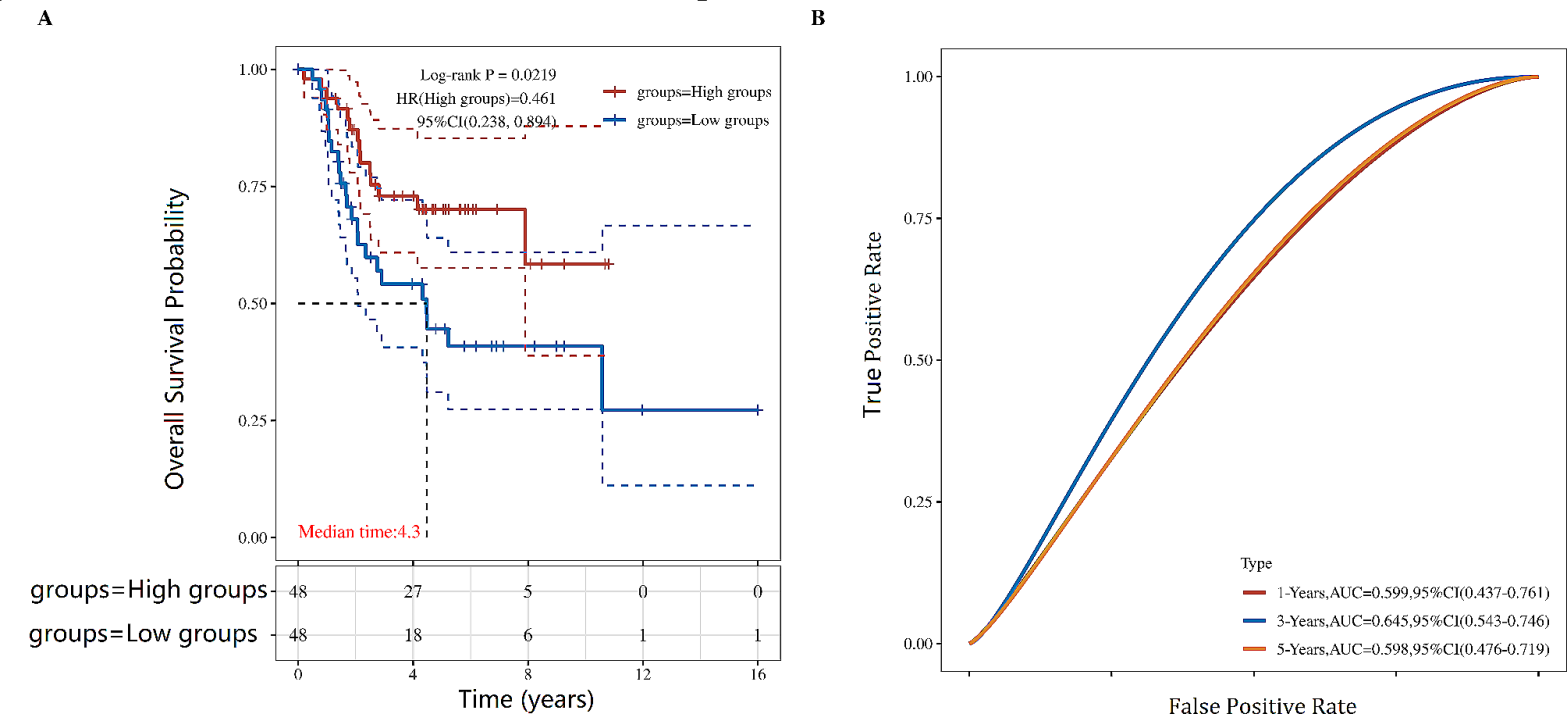
Kaplan-Meier survival analysis of GPR65 expression of 98 OS patients in the TARGET database. (**A**) Compared to OS patients with low GPR65 expression, the high GPR65 expression group had significantly higher 3-year and 5-year survival rates (*P* < 0.0219, HR = 0.461). (**B**) The model demonstrated good ability to discriminate, which was stable when tested in the test set (AUC = 0.645)

Through LinkedOmic (http://linkedomics.org/login.php) analysis of the Kaplan-Meier survival curves of 98 OS patients in the TARGET database based on GPR65 high and low expression (Median). The results showed that compared to patients with low GPR65 expression of OS, the high GPR65 expression group had significantly higher 3-year and 5-year survival rates (*P* < 0.0219, HR = 0.461, Fig. 5A). The ability to predict prognosis were determined by receiver operating characteristic curve (ROC), the results showed the model demonstrated good ability to discriminate, which was stable when tested in the test set (AUC = 0.645, Fig. 5B).

To explore the predictive value of GPR65 for the prognosis of OS patients, we conducted a COX proportional risk model analysis on 97 OS patients. Univariate COX analysis found a significant correlation between GPR65 expression, metastasis, FRT, histological response, EFS, and OS patients’ survival (*P* < 0.05, HR < 1). Further multivariate COX regression analysis of the above indicators revealed a consistent trend between GPR65 expression and metastasis, FRT, histological response, and EFS univariate COX regression (Table 2). These findings revealed that the expression of GPR65 is a favorable prognostic factor for overall survival in patients with OS.

**Table 2:** Univariate and multivariate analysis of prognostic parameters in TARGET database overall survival

Variable	Univariate analysis	P-value	Multivariate analysis	P-value
	HR (95% CI)		HR (95% CI)	
GPR65 expression	0.775(0.623–0.965)	0.022	1.666(1.019–2.724)	0.042
Metastasis	0.25(0.13–0.482)	0	0.235(0.042–1.306)	0.098
FRT	0.998(0.997-0.0999)	0.001		
Histologic response	0.2(0.045–0.88)	0.033	0.23(0.017–3.158)	0.271
EFS	0.997(0.996–0.998)	0	0.994(0.989–0.999)	0.009

### Lower GPR65 expression is necessary for osteosarcoma cell growth

**Fig. 6:**
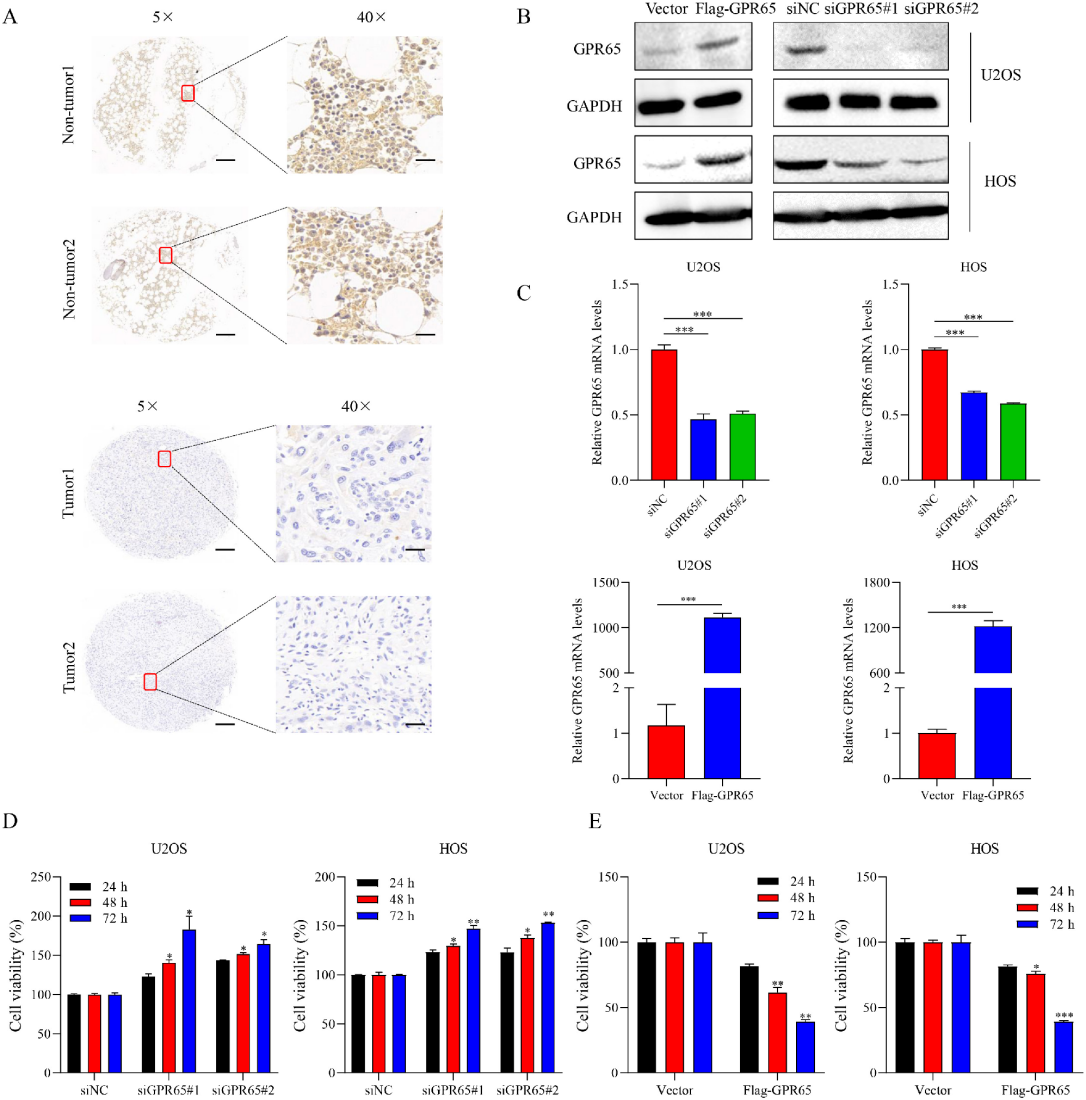
GPR65 is downregulated in OS tumor tissues. (**A**) Representative images of IHC staining for GPR65 in a clinical sample tissue microarray. Magnification: 5თ(scale bar: 200 μm) and 40თ(scale bar: 20 μm). *n* = 15 per group. (**B-C**) Western blot (**B**) or qRT-PCR (**C**) analysis of GPR65 expression in U2OS and HOS cells transfected with Flag-GPR65 or siRNAs against GPR65 (siGPR65). (**D-E**) MTT assay detected the viability of OS cells with or without GPR65 overexpression (**D**) or knockdown (**E**). ^*^p < 0.05 significantly different from control (siNC or Vector) group

**Fig. 7:**
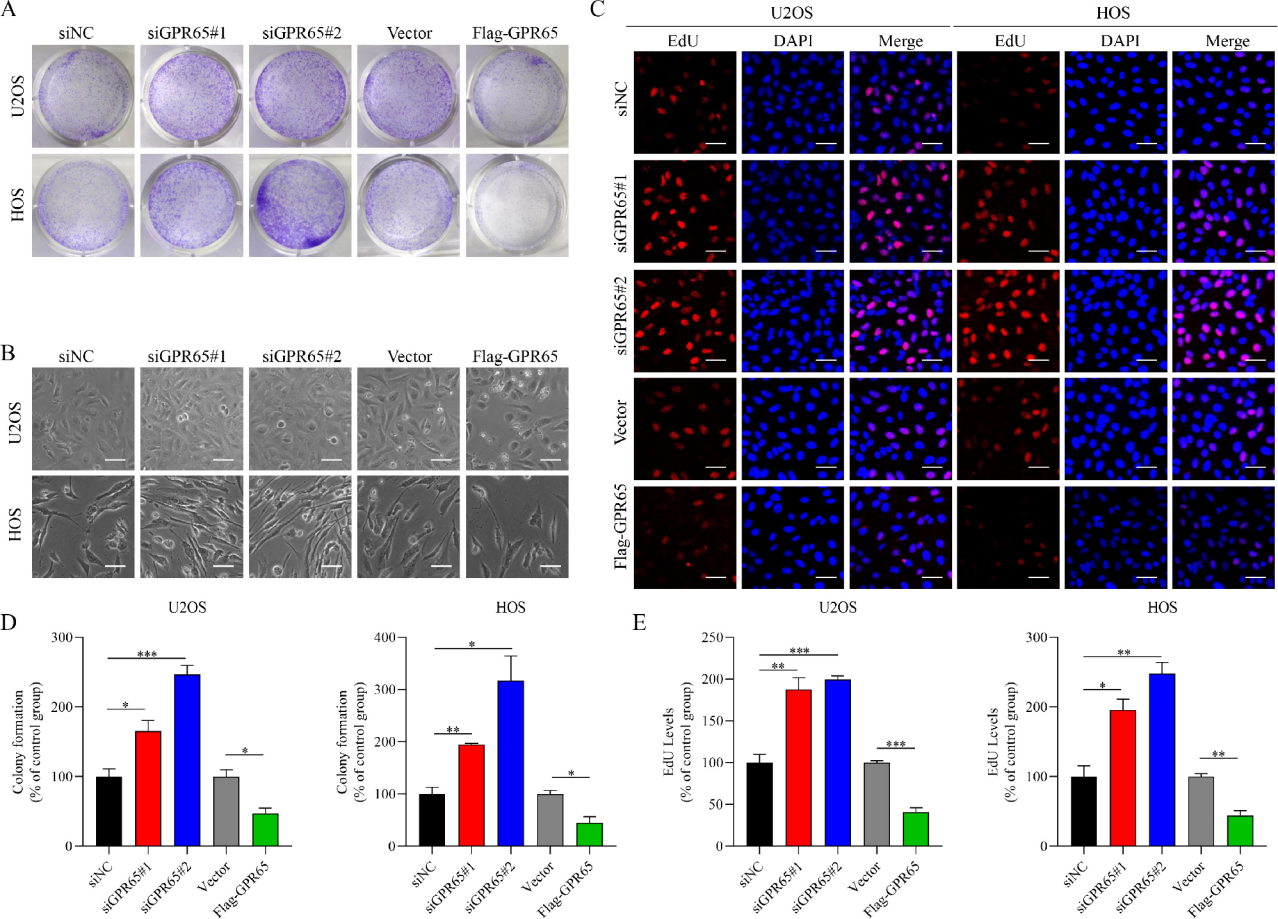
Low expression of GPR65 is necessary for the survival of OS cells. (**A**) The effect of GPR65 on clone formation in U2OS and HOS cells. (**B**) The effect of GPR65 on cell morphology of U2OS and HOS. Scale bars: 200 μm. (**C**) Representative images of the EdU (red) experiment of different GPR65 expression levels. Nuclei were stained with DAPI (blue). Scale bars: 200 μm. (**D**) Data of numbers of clone formation per field. (**E**) Data of the red fluorescence intensity of EdU. Data are mean ± SEM from three independent experiments. *P < 0.05, **P < 0.01, ***P < 0.001 compared with the control group

To further clarify the role of GPR65 in the development of osteosarcoma, we first examined the expression of GPR65 in tissue microarray of osteosarcoma and normal bone tissue (Fig. 6A). Consistent with the database results, the expression level of GPR65 in osteosarcoma patients was significantly lower than that in normal bone tissue. At the same time, we conducted confirmatory experiments in human osteosarcoma cell lines U2OS and HOS. We confirmed the expression effect of GPR65 overexpressed plasmid and siRNA (Fig. 6B-6C). The MTT assay indicated a considerable decrease in cell viability after GPR65 overexpression compared with the empty plasmid group, while silencing GPR65 expression could enhance the proliferation ability of U2OS and HOS cells (Fig. 6D). The same phenomenon was observed in colony formation experiments (Fig. 7A and 7D). It was found that the number of GPR65 cells increased significantly after knocking down GPR65 expression, and became long spindle shape. The opposite was true when GPR65 was highly expressed (Fig. 7B). Similarly, the EdU incorporation assay showed that U2OS and HOS cell proliferation was notably promoted in cells after GPR65 silencing. However, the cell growth was inhibited when GPR65 was highly expressed (Fig. 7C and 7E). Taken together, GPR65 expression is decreased in osteosarcoma tissues, and knocking-down GPR65 expression in osteosarcoma cells significantly promotes cell proliferation.

### Silencing GPR65 enhances osteosarcoma cells invasiveness

**Fig. 8:**
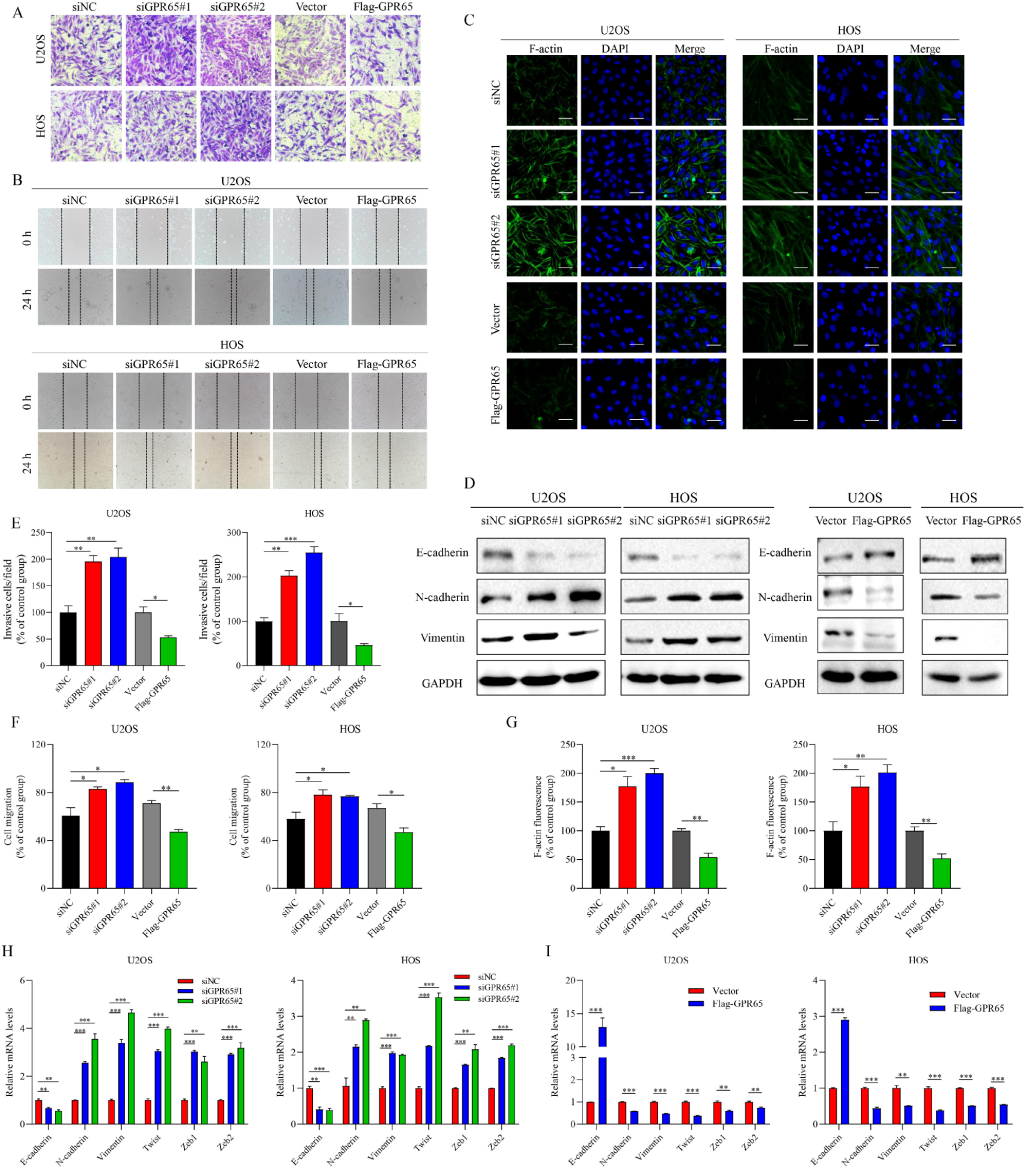
GPR65 is negatively correlated with the migration of OS cells. (**A**) Typical images of U2OS and HOS cells crossing the non-matrigel-coated membrane (transwell assay). (**B**) Typical images of U2OS and HOS cells at initial (0 h) and final position (24 h) (wound-healing assay). (**C**) U2OS and HOS cells were transfected with siNC and siGPR65 or Vector and Flag-GPR65 plasmids, followed by immunofluorescence assay to observe F-actin (green). Nuclei were stained with DAPI (blue). Scale bars: 200 μm. (**D**) The changes of EMT markers protein levels after GPR65 knockdown or overexpression. (**E**) Data of numbers of invasive cells per field. (**F**) Data of relative migration index. (**G**) Data of the green fluorescence intensity of F-actin. (**H-I**) qRT–PCR showed the changes in EMT markers after GPR65 knockdown or overexpression. Data are mean ± SEM from three independent experiments. *P < 0.05, **P < 0.01, ***P < 0.001 compared with the control group

Given the changes in cell morphology of U2OS and HOS cells with different GPR65 expression, we hypothesized that GPR65 may be involved in the invasion and metastasis of osteosarcoma cells. To test this idea, we conducted a series of functional experiments to verify it. In transwell assay, the elevated number of invasive cells per field in GPR65-knockdown group was indeed increased (Fig. 8A and 8E). Meanwhile, wound healing assays indicated that GPR65-silencing cells had extremely increase cell mobility compared with siNC group cells, suggesting accelerated cell migration and invasion ability, whereas GPR65 overexpression could decrease cell invasion and migration capacity (Fig. 8B and 8F). In addition, we observed a noticeable increase in F-actin formation in both U2OS and HOS cells after endogenous GPR65 expression was silenced, while forced GPR65 expression restrained the growth of F-actin (Fig. 8C and 8G). Similar results were also obtained in the EMT-related marker expression (Fig. 8D, 8H and 8I). Collectively, these findings demonstrated the pivotal role of GPR65 in cytoskeletal reorganization that facilitates tumor cell migration.

### Analysis of downstream gene and signaling pathway regulating GPR65 in osteosarcoma cells

**Fig. 9:**
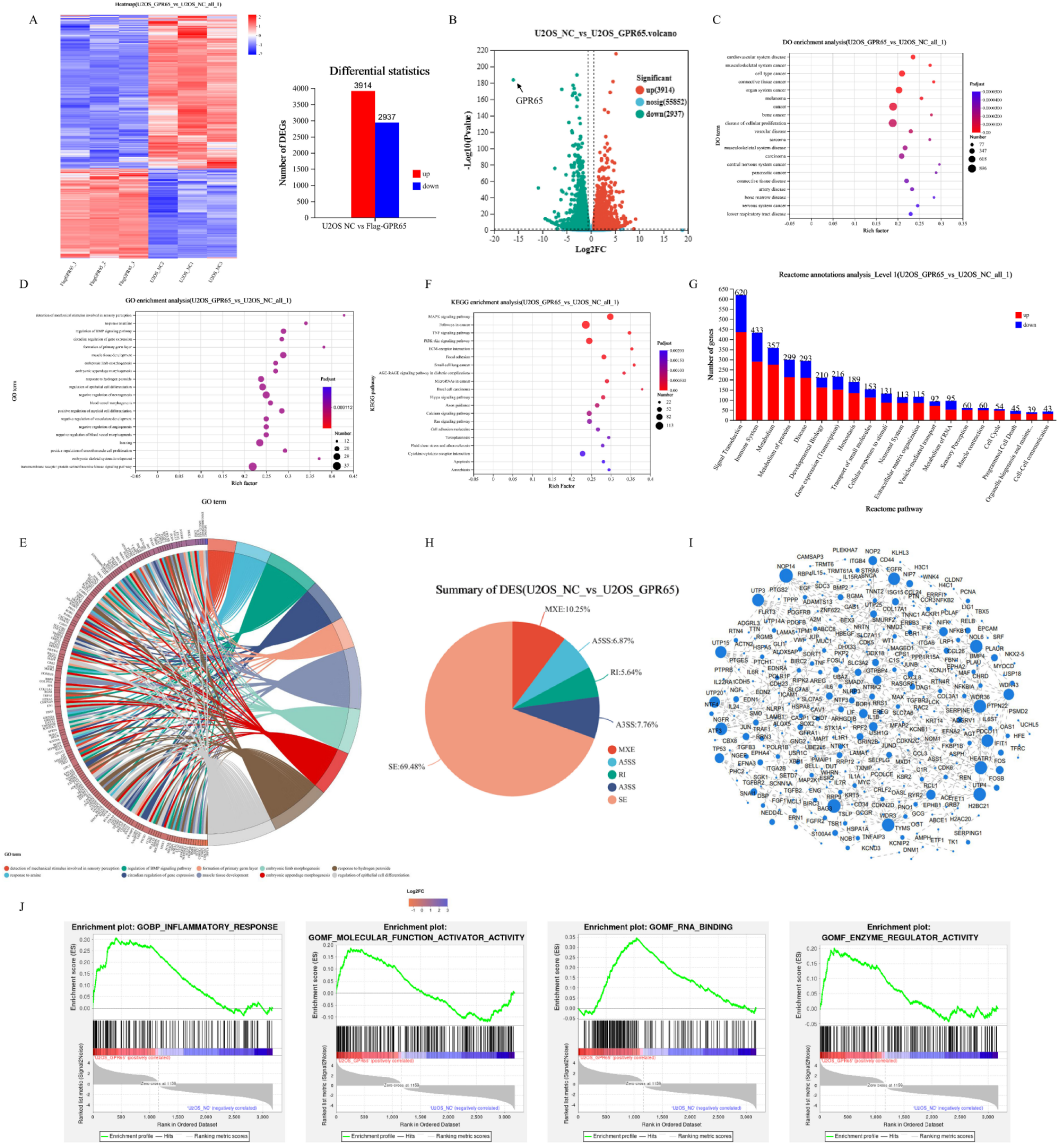
Analysis of downstream gene and signaling pathway regulating GPR65 in osteosarcoma cells. (**A-B**) Heatmap and Volcano plot shows differentially expressed genes in U2OS cells with or without GPR65 overexpression. (**C-D**) DO and GO enrichment analysis of DEGs in U2OS cells (Flag-GPR65 vs. NC). (**E**) Enriched chordal diagram of DEGs in U2OS cells. (**F-G**) KEGG enrichment analysis and Reactome annotations analysis of DEGs in U2OS cells. (**H**) GPR65-mediated Alternative Splicing (AS) events. (**I**) Protein-protein interactions (PPI) between DEGs in U2OS cells. (**J**) GSEA enrichment analysis of DEGs

To investigate the potential mechanism of GPR65 in osteosarcoma cells, unbiased transcriptome analysis was performed by RNA sequencing (RNA-seq) on samples of U2OS cells with or without GPR65 overexpression, and all the analyses were conducted by the Majorbio Cloud Platform (www.majorbio.com). Based on the quantitative expression results, inter-group differential gene analysis was performed to obtain differentially expressed genes (DEGs) between the two groups. The difference analysis software was DESeq2, and the screening threshold was |log2FC| ≥ 0.585 & *P* value ≤ 0.05. According to cluster analysis and volcano map results, a total of 6851 DEGs were identified, among which 2310 genes were upregulated and 1348 genes were downregulated (Fig. 9A and 9B). Meanwhile, disease ontology (DO) enrichment analysis showed that DEGs were closely related to cancers, especially orthopedic cancers (Fig. 9C). The results of GO enrichment analysis and enrichment chord diagram demonstrated that the DEGs after up-regulation of GPR65 were involved in muscle tissue development, regulation of epithelial cell differentiation, positive regulation of myeloid cell differentiation and other processes, revealing the important role of GPR65 in bone marrow disease occurrence (Fig. 9D and 9E). And the KEGG enrichment analysis indicated the DEGs were highly relevant to MAPK signaling pathway, focal adhesion and PI3K-Akt pathway (Fig. 9F). In addition, Reactome annotations analysis revealed that differential genes are closely related to signal transduction, immune system and metabolism (Fig. 9G).

To further investigate the regulatory mechanisms of GPR65 in osteosarcoma, thousands of GPR65-mediated Alternative Splicing (AS) events were defined by AS analysis of rMARTs. AS shown in the Fig. 9H, skip exons (SE) was the dominant type of AS events, accounting for 69.48%, followed by mutually exclusive exons (MXE) (10.25%) and alternative 3 ‘splice site (A3SS) (7.76%). It’s indicated that GPR65 principally modulated SE. Moreover, protein-protein interactions (PPI) between DEGs were predicted using a STRING database, involving a total of 285 nodes and 764 edges, and most of these proteins were involved in antiviral and immune processes (Fig. 9I). GSEA further indicated that these DEGs were closely related to the inflammatory response, molecular function activator activity, RNA binding and enzyme regulator activity (Fig. 9J). All these results confirmed that GPR65 plays an important role in multiple processes of osteosarcoma development. High expression of GPR65 predicted the enhancement of immune response, anti-inflammatory and anti-tumor ability.

## Discussion

This study analyzed 97 patients with OS from TAEGET database. The data records of this OS patient were relatively complete, and four patients with missing clinical data were deleted. It is well known that compared with other types of cancer, OS has a relatively low incidence rate. Therefore, in terms of the number of cases, it is already a relatively large number of OS cases to study 97 patients with OS. OS is more common in adolescents, and the younger the age, the higher grade of malignancy and the greater the harm.

About 25% of patients with OS have detectable metastases, most commonly in the lungs [18]. Previous or potential distant metastases lead to a high recurrence rate. At present, the common treatment options such as radiotherapy, chemotherapy and surgery have not achieved satisfactory clinical results [19]. Cellular immunotherapy, stem cell therapy, and targeted therapy have been used in patients with recurrent OS in recent years [20, 21]. Tumor immunotherapy plays an anti-tumor role by stimulating and enhancing the immune response of the body. Compared with chemotherapy, radiotherapy and targeted therapy, it has become another important way of anti-tumor therapy, with significant clinical efficacy and advantages [22]. CD8 + cytotoxic T lymphocytes (CTL), CD4 + T cells, NK cells and NKT cells all play critical roles in tumor immunity, while humoral immunity may not only inhibit tumor growth but also enhance it [23]. Researchers have devised various strategies to boost the immune system in recent years based on tumor immune response studies. It has been found that OS cells can establish a local microenvironment conducive to tumor growth, drug resistance and metastasis by controlling the recruitment and differentiation of immune-infiltrating cells [24].

G-protein-coupled receptors (GPCRS) are the largest superfamily of transmembrane proteins encoded by the human genome, mediating most cellular responses to external stimuli, including light, odor, ions, hormones, and growth factors [25]. GPR65 is a pH-sensing G protein-coupled receptor that acts as a key innate immune checkpoint in the human tumor microenvironment, inhibiting the release of inflammatory factors and inducing significant upregulation of tissue repair genes [11]. Pathios has developed PTT-3213, a small molecule inhibitor of GPR65 that significantly increases CD8 + T cells and natural killer T (NKT) cells in the tumor microenvironment. It can synergize with PD1 antibody to produce better efficacy in mouse MC38 tumor models. GPR65 can be activated by protons when the pH value is lower than 7.2, leading to the increase of cAMP and the activation of A (RhoA), a member of the Ras homologous gene family [26]. Among our 97 patients with OS, grouping analysis based on the average GPR65 expression of 6.884 (close to 7.2) for high and low expression is more scientific and reasonable. our study found that GPR65 is low expressed in young patients, and the older the age, the higher the expression of GPR65. Moreover, this study found that GPR65 expression was lower in metastatic OS patients, while it was higher in non-metastatic OS patients. High expression of GPR65 in OS patients with high overall survival rate. This means that high expression of GPR65 indicates a good prognosis for patients with OS. Further analysis of the molecular role and mechanism of GPR65 in OS patients revealed that GPR65 is mainly associated with tumor immunity in patients. Surprisingly, our study is completely different from other studies, where the higher the expression of GPR65, the worse the malignancy and prognosis of cancer [27, 28]. Therefore, our study suggests that GPR65 as a new immune checkpoint for immune checkpoint inhibitor anti-tumor therapy (ICI-therapy) is controversial, and at least not applicable to some malignant tumors, including OS. Further ScRNA sequencing analysis showed that GPR65 was highly expressed in CD4 + cells and macrophages in the microenvironment of OS. This indicates that GPR65 is involved in the tumor immune regulatory response of OS.

Further experiments have verified the above information results. The expression of GPR65 is significantly decreased in osteosarcoma tissues. Silencing the expression of GPR65 in osteosarcoma cells U2OS and HOS can promote the proliferation and invasion process, while overexpression of GPR65 can inhibit this process. Further RNA-seq results showed that high expression of GPR65 in U2OS cells can induce changes in immune system, metabolism, and signaling processes, and exert tumor inhibition through MAPK and PI3K/AKT signaling pathways. Among many signaling pathways, MAPK signaling pathway plays a particularly important role in cell proliferation, differentiation, apoptosis, angiogenesis and tumor metastasis [29]. There is reported that dioscin induces OS cell apoptosis by upregulating ROS-mediated P38 MAPK signaling [30], and Fan’s research also showed that siglec-15 promotes tumor progression in OS via DUSP1/MAPK pathway [31]. Similarly, the role of PI3K/AKT pathway in reversing drug resistance in OS is confirmed by the examination of some reports [32, 33]. However, how GPR65 functions through MAPK and PI3K/AKT pathways in OS not yet clear, and we will address this issue in future studies.

From the above discussion, the conclusion can be reached that GPR65 cannot be used as an ICI target for OS immunotherapy, but rather as a favorable prognostic factor for overall survival in OS patients. The suppression of immune escape and inhibition of proliferation may be a key pathway for GPR65 to participate in the progression of OS.

### Electronic supplementary material

Below is the link to the electronic supplementary material.

Supplementary Material 1: The expression of GPR65 in different cancer tissues and normal tissues

Supplementary Material 2: Clinical characteristics of 97 patients with OS in TARGET database

## Data Availability

Osteosarcoma cell lines U2OS and HOS were obtained from Procell Life Science&Technology Co.,Ltd (Wuhan, China).

## Abbreviations

OS: Osteosarcoma
FRT: Time to First Relapse in Days
PSP: Primary Site Progression
MS: Metastasis Status
FE: First Event
TME: Tumor microenvironment
ECM: Extracellular matrix
BP: Biological process
CC: Cell component
MF: Molecular function
GEPIA: Gene Expression Profiling Interaction Analysis
KEGG: Kyoto Encyclopedia of Genes and Genomes
GEO: Gene Expression Omnibus
FBS: Fetal bovine serum
GSVA: Gene set variation analysis
GO: Gene ontology
ROC: Receiver operating characteristic curve
A3SS: Alternative 3 ‘splice site
RNA-seq: RNA sequencing
DEGs: Differentially expressed genes
DO: Disease ontology
AS: Alternative Splicing
SE: Skip exons
MXE: Mutually exclusive exons
PPI: Protein-protein interactions
