# Supplementary figures and images for: What role does GPR65 play in the progression of osteosarcoma? Its mechanism and clinical significance
Source: Cancer Cell Int. 2024 Jan 13;24:31. doi: 10.1186/s12935-024-03216-5 (PMC10788037; doi:10.1186/s12935-024-03216-5)

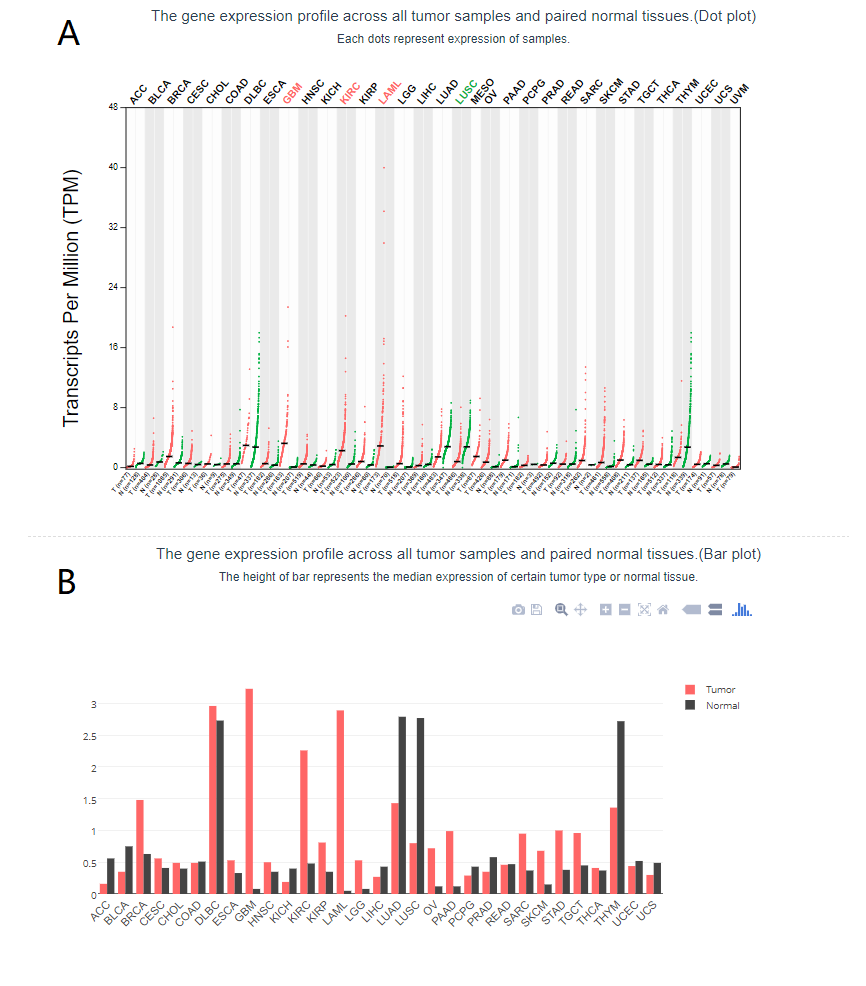

Supplement: Supplementary file 1 — Supplementary Material 1: The expression of GPR65 in different cancer tissues and normal tissues [file 12935_2024_3216_MOESM1_ESM.png]
